# Supplementary material for: Hypoxia Is Associated with Increased Immune Infiltrates and Both Anti-Tumour and Immune Suppressive Signalling in Muscle-Invasive Bladder Cancer
Source: Int J Mol Sci. 2023 May 18;24(10):8956. doi: 10.3390/ijms24108956 (PMC10219458; doi:10.3390/ijms24108956)
Supplement: Supplementary file 1 [file ijms-24-08956-s001.zip › ijms-2340374-supplementary.pdf]

Supplementary Table S1. Details of the antibodies used for ChIPseq experiments.

Target	Raised in	Concentration	Supplier	Code
HIF-1α	Rabbit	1mg/mL	Abcam	Ab2185
HIF-2α	Rabbit	1mg/mL	Abcam	Ab199
HIF-1β	Mouse	1mg/mL	Novus Biologicals	H1beta234
CTCF	Rabbit	1mg/mL	Diagenode	C01010170
IgG control	Rabbit	1mg/mL	Millipore	12-370

Supplementary Table S2. Number of peaks according to different filtering parameters for each sample.

Oxygen	Sample	AllSigPeaks	Protein Coding	Near TSS	Protein Coding and near TSS
0.1%	HIF-1α	30486	23536	7514	6801
	HIF-1β	4857	3539	975	848
	HIF-2α	72520	48438	3435	2883
1%	HIF-1α	32123	24728	6712	6043
	HIF-1β	5963	4291	990	847
	HIF-2α	36505	24713	1974	1612

Supplementary Table S3. GO terms filtered by the search term “immun” significantly enriched under 1% hypoxia.

Term	ID	Ont	N	DE	P.DE
somatic diversification of immune receptors via germline recombination within a single locus	GO:0002562	BP	66	7	0.05
neutrophil mediated immunity	GO:0002446	BP	501	43	<0.001
neutrophil activation involved in immune response	GO:0002283	BP	490	42	<0.001
myeloid leukocyte mediated immunity	GO:0002444	BP	555	46	<0.001
myeloid cell activation involved in immune response	GO:0002275	BP	549	43	0.003
leukocyte activation involved in immune response	GO:0002366	BP	716	51	0.01
cell activation involved in immune response	GO:0002263	BP	720	51	0.01

Ont is the gene ontology term, BP = biological process. N = number of genes in the GO term. DE = number of differentially expressed genes from dataset present in the GO term. P.DE = p value for over representation of the GO term in the set.

Supplementary Table S4. GO terms filtered by the search term “immun” significantly enriched under 0.2% hypoxia.

Term	ID	Ont	N	DE	P.DE
somatic recombination of immunoglobulin genes involved in immune response	GO:0002204	BP	51	15	0.01
somatic diversification of immunoglobulins involved in immune response	GO:0002208	BP	51	15	0.01
somatic recombination of immunoglobulin gene segments	GO:0016447	BP	56	15	0.02
immunoglobulin production involved in immunoglobulin-mediated immune response	GO:0002381	BP	61	15	0.05
somatic diversification of immune receptors via germline recombination within a single locus	GO:0002562	BP	66	16	0.05
somatic diversification of immunoglobulins	GO:0016445	BP	66	16	0.05
neutrophil mediated immunity	GO:0002446	BP	501	100	0.006
neutrophil activation involved in immune response	GO:0002283	BP	490	95	0.02
myeloid leukocyte mediated immunity	GO:0002444	BP	555	105	0.02

Ont is the gene ontology term, BP = biological process. N = number of genes in the GO term. DE = number of differentially expressed genes from dataset present in the GO term. P.DE = p value for over representation of the GO term in the set.

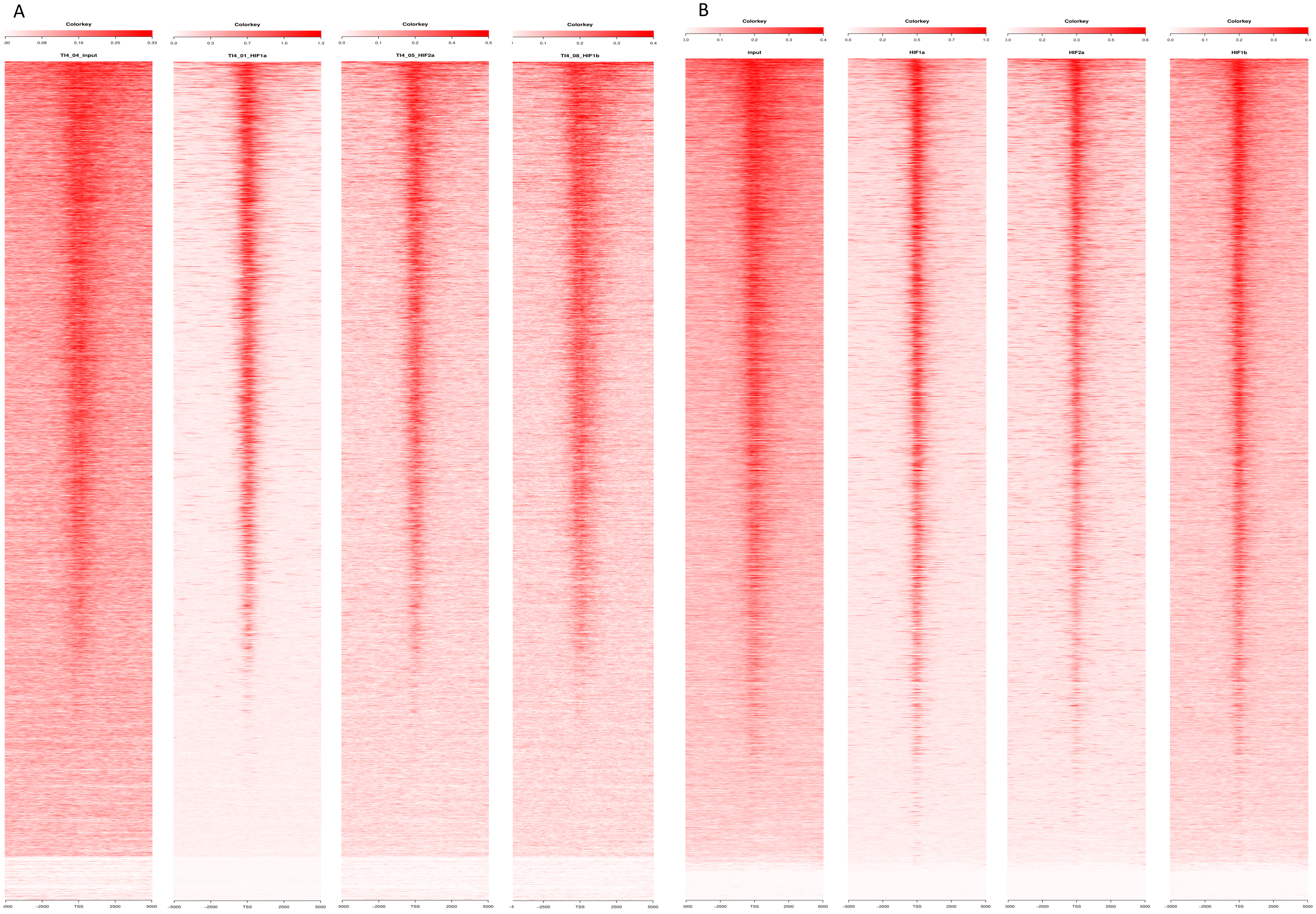
A
Colorkey
TI4_04_input
Colorkey
TI4_01_HIF1a
Colorkey
TI4_05_HIF2a
Colorkey
TI4_08_HIF1b
B
Colorkey
input
Colorkey
HIF1a
Colorkey
HIF2a
Colorkey
HIF1b
−2500
TSS
2500
5000

C D

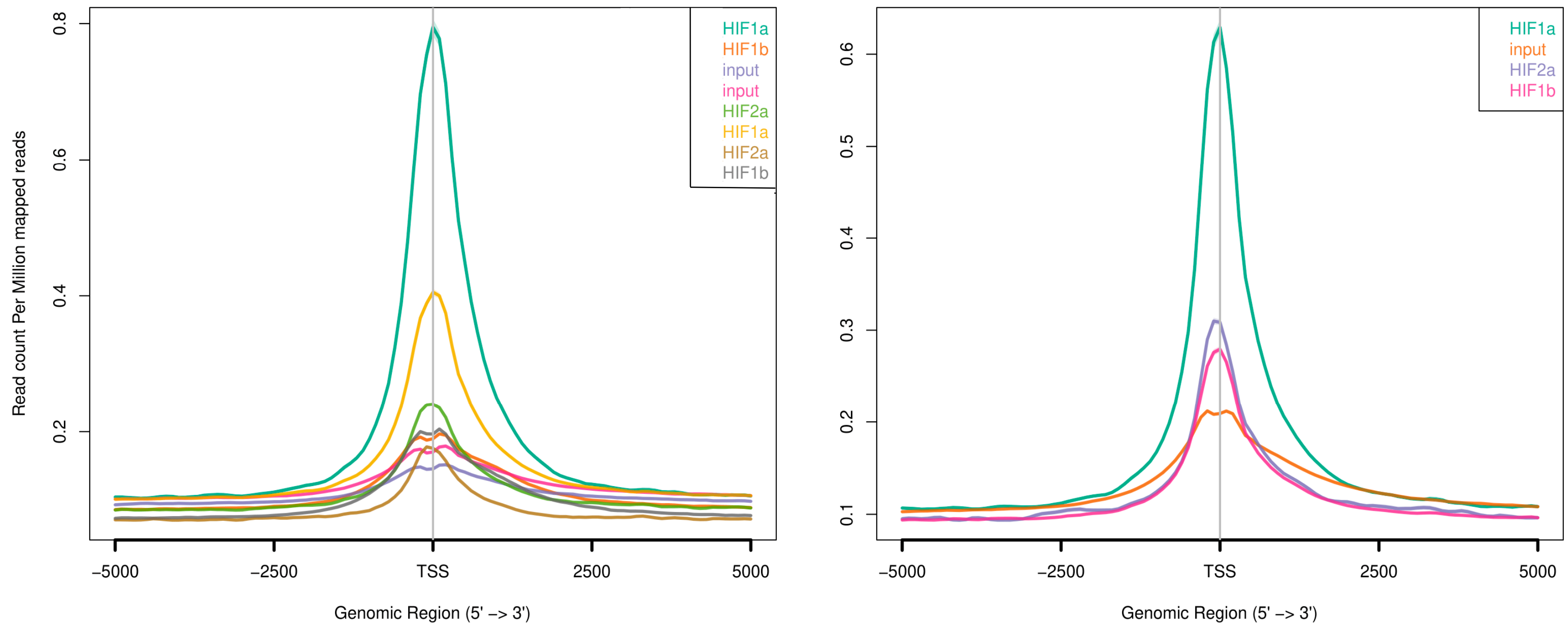

Supplementary Figure S1. Heatmaps of signal intensities for T24 cells cultured under **A**) 1% O_2 and **B**) 0.1% O_2 and enrichment of mapped reads around the transcriptional start site for each sample under **C**) 1% O_2 and **D**) 0.1% O_2.

Binding sites for each sample were identified by MACS peak caller and ordered on the y-axis according to signal intensity. Heatmaps show the signal (read counts per million mapped reads) expressed as colour intensity (darker colour = higher signal). The x-axis shows genomic region of mapped reads at the transcriptional start sites and across flanking ± 5kb regions. Graphs were generated using R package "ngsplot". Left to right: input DNA, HIF-1α, HIF-2α and HIF-1β ChIP. The signal reads are expressed as counts per million mapped reads (y-axis) across flanking ± 5kb regions (x-axis). Graphs were generated using package "ngplpots". 1% O_2 data were generated in duplicate, 0.1% O_2 data were single replicates.

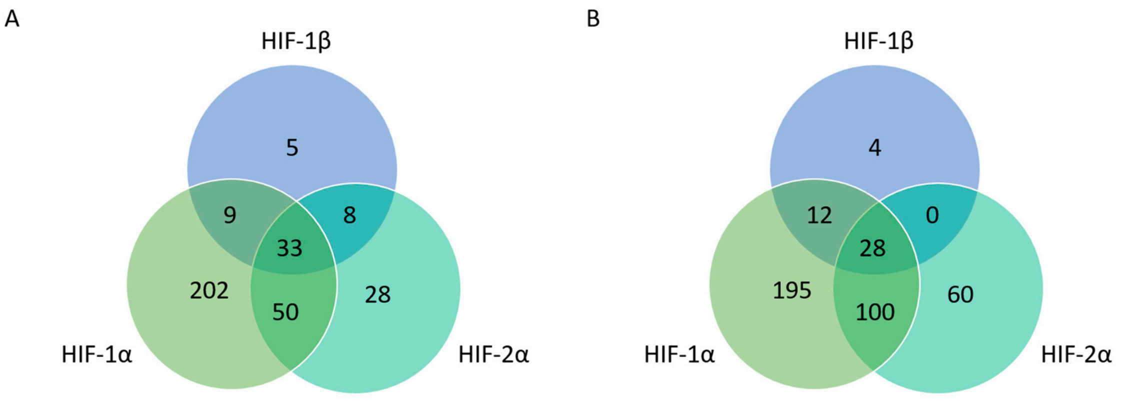

Supplementary Figure S2. Venn diagrams showing the number of immune response genes overlapping between the HIF subunits for **A**) 1% oxygen and **B**) 0.1% oxygen.
The most lenient filtering parameter, all significant peaks, for each sample under both oxygen concentrations was used. Immune response genes were defined using those annotated as “immune response” from the EBI QuickGO resource.

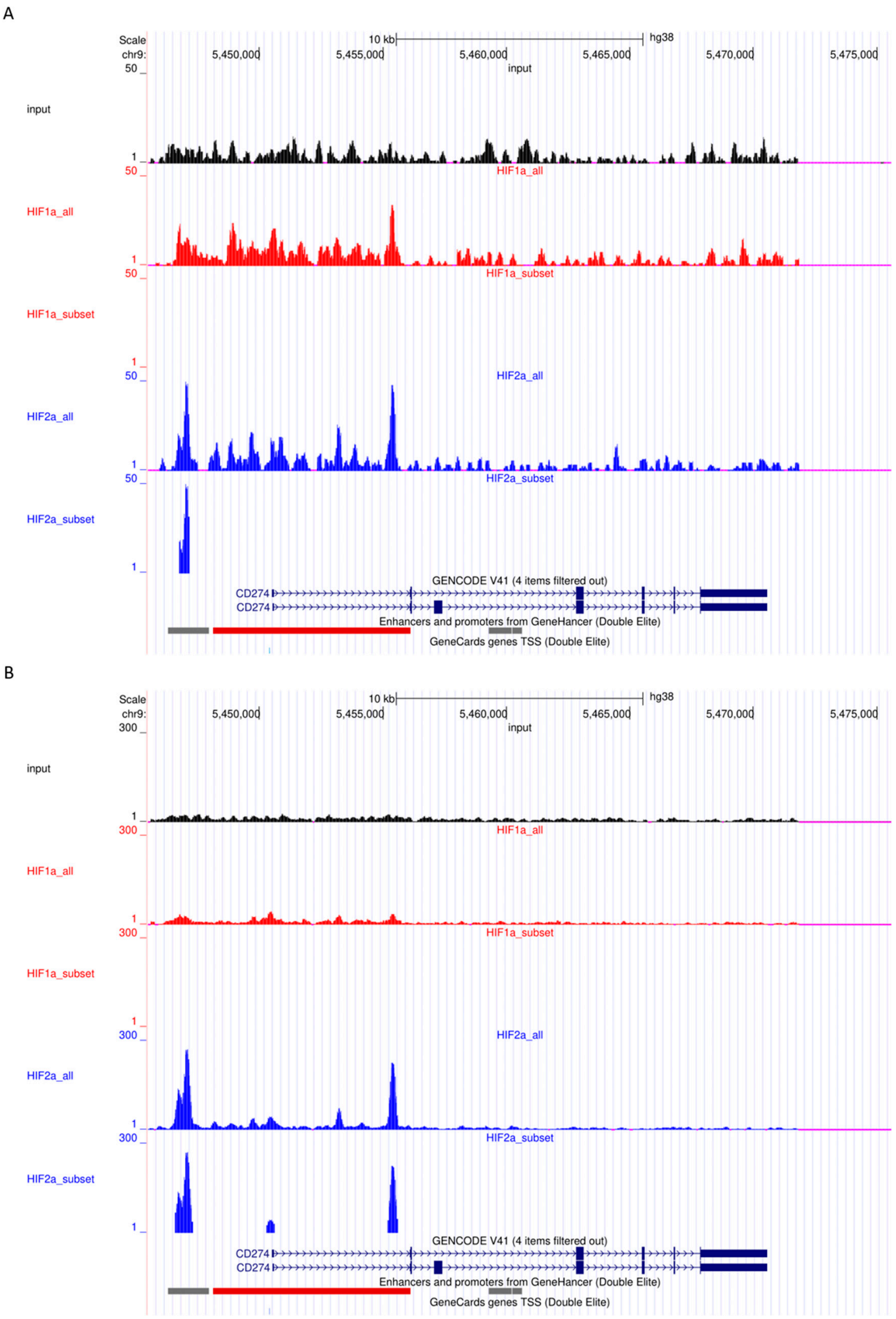

Supplementary Figure S3. ChIPseq tracks for the CD274 gene (encoding the PD-L1 protein) shown using the UCSC genome browser for **A**) 1% and **B**) 0.1% samples.

Representative tracks are displayed from top to bottom in each image for input: HIF-1α (no filtering), HIF-1α (filtering), HIF-2α (no filtering), and HIF-2α (filtering). The track shown at the bottom annotates known enhancers and promoters from the GeneHancer database where: grey = enhancer and red = enhancer/promoter. The corresponding peaks above the CD274 enhancer region for the HIF-2α tracks demonstrate the enrichment of HIF-2α in this genomic region in T24 cell line.

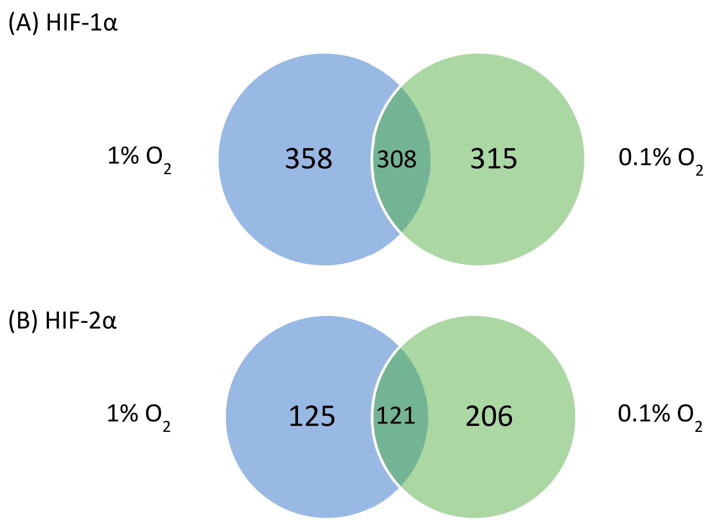

Supplementary Figure S4. Venn diagrams showing the number of unique immune response genes overlapping between different oxygen concentrations for **A**) HIF-1α and **B**) HIF-2α.
Genes unique to each subunit are shown using the most lenient filtering parameter, all significant peaks, for each sample under both oxygen concentrations. Immune response genes were defined using those annotated as "immune response" from the EBI QuickGO resource.

A

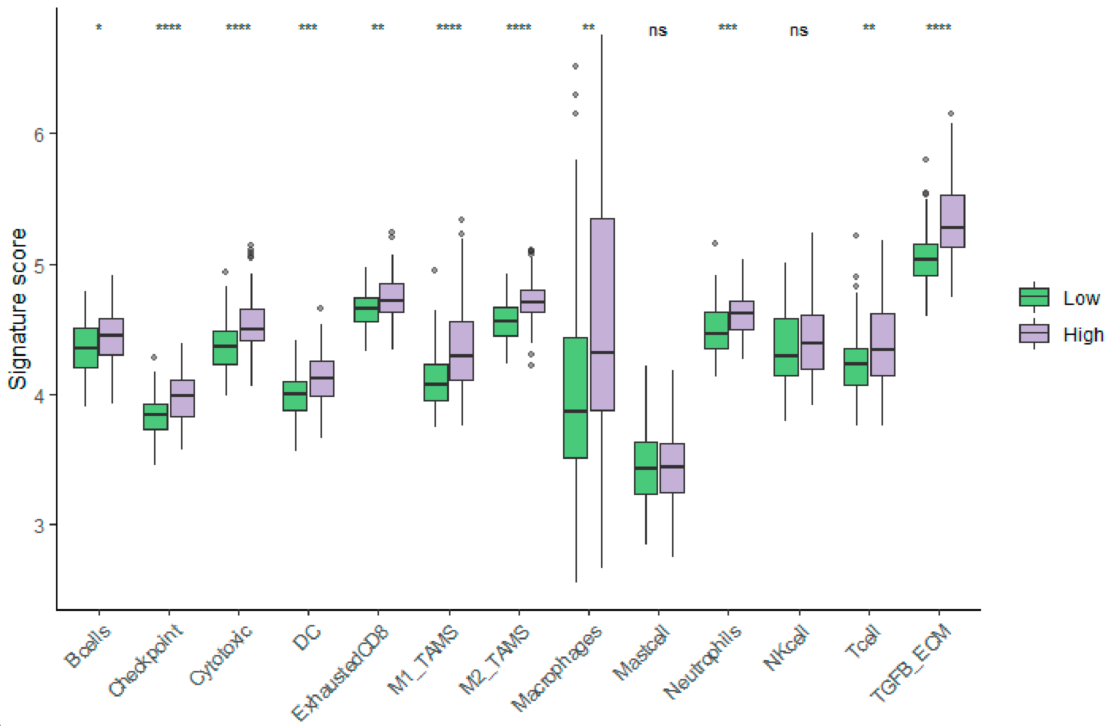

B

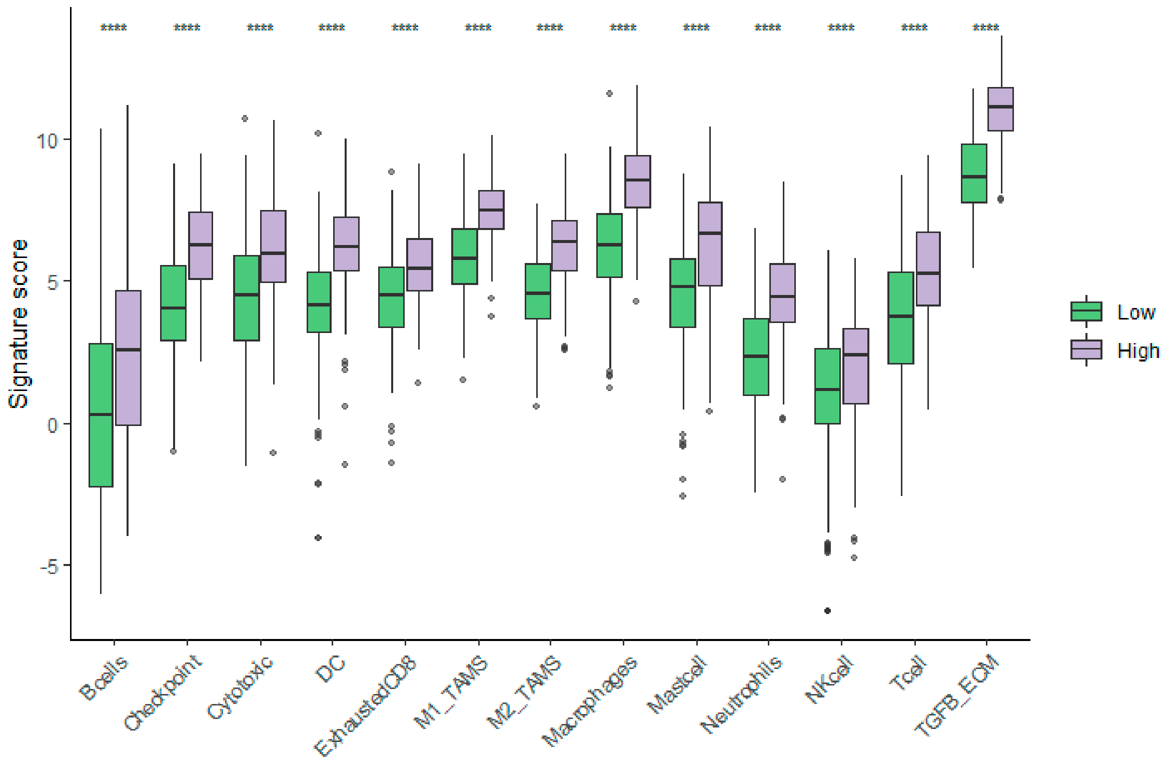

Supplementary Figure S5. Boxplots showing the score of immune-related signatures according to hypoxia score for **A**) BCON and **B**) TCGA. Signature scores were calculated using the mean expression of the genes in the signature. Hypoxia status was stratified by the median hypoxia score of the cohort. Statistics are p values from t tests with p values represented as: ns = not significant, *<0.05, **<0.01, ***<0.001, ****<0.0001.
